# Dynamic Changes of Heart Failure Biomarkers in Response to Parabolic Flight

**DOI:** 10.3390/ijms21103467

**Published:** 2020-05-14

**Authors:** Peter Jirak, Bernhard Wernly, Michael Lichtenauer, Vera Paar, Marcus Franz, Thorben Knost, Thaer Abusamrah, Malte Kelm, Johanna M. Muessig, Nana-Yaw Bimpong-Buta, Christian Jung

**Affiliations:** 1 Department of Internal Medicine II, Division of Cardiology, Paracelsus Medical University of Salzburg, 5020 Salzburg, Austria; p.jirak@salk.at (P.J.); b.wernly@salk.at (B.W.); m.lichtenauer@salk.at (M.L.); v.paar@salk.at (V.P.); 2 Department of Internal Medicine I, Jena University Hospital, Friedrich Schiller University Jena, 07743 Jena, Germany; Marcus.Franz@med.uni-jena.de; 3 Division of Cardiology, Pulmonology, and Vascular Medicine, Medical Faculty, University Duesseldorf, 40225 Duesseldorf, Germany; Thorben.Knost@med.uni-duesseldorf.de (T.K.); Thaer.Abusamrah@med.uni-duesseldorf.de (T.A.); Malte.Kelm@med.uni-duesseldorf.de (M.K.); Johanna.Muessig@med.uni-duesseldorf.de (J.M.M.); 4 Division of Cardiology and Rhythmology, Evangelical Hospital Hagen-Haspe, 58135 Hagen, Germany; nanaybbb@yahoo.com

**Keywords:** space medicine, parabolic flight, microgravity, weightlessness, biomarkers, heart failure

## Abstract

Background: we aimed at investigating the influence of weightlessness and hypergravity by means of parabolic flight on the levels of the heart failure biomarkers H-FABP, sST2, IL-33, GDF-15, suPAR and Fetuin-A. Methods: 14 healthy volunteers (males: eight; mean age: 28.9) undergoing 31 short-term phases of weightlessness and hypergravity were included. At different time points (baseline, 1 h/24 h after parabolic flight), venous blood was drawn and analyzed by the use of ELISA. Results: sST2 evidenced a significant decrease 24 h after parabolic flight (baseline vs. 24, *p* = 0.009; 1 h vs. 24 h, *p* = 0.004). A similar finding was observed for GDF-15 (baseline vs. 24 h, *p* = 0.002; 1 h vs. 24 h, *p* = 0.025). The suPAR showed a significant decrease 24 h after parabolic flight (baseline vs. 24 h, *p* = 0.1726; 1 h vs. 24 h, *p* = 0.009). Fetuin-A showed a significant increase at 1 h and 24 h after parabolic flight (baseline vs. 24 h, *p* = 0.007; 1 h vs. 24 h, *p* = 0.04). H-FABP and IL-33 showed no significant differences at all time points. Conclusion: Our results suggest a reduction in cardiac stress induced by exposure to gravitational changes. Moreover, our findings indicate an influence of gravitational changes on proliferative processes and calcium homeostasis.

## 1. Introduction

Human space missions have experienced a revival in recent years. While the National Aeronautics and Space Administration (NASA) aims for a manned mission to Mars in co-operation with other space agencies, suborbital commercial space flights are about to enter the private sector. Given the expected rise in manned space flights, space medicine is gaining major importance as a necessity for safe and successful missions in the future [1].

In the absence of gravity, the human body undergoes multiple adaptational processes, which have been analyzed in previous studies [2]. Above all, changes in the cardiovascular system have been reported [3]. Cardiovascular causes are involved in the majority of medical complications in human space missions and represent an important target in space medicine [3,4]. In this regard, the most significant change is an increase in cardiac output of up to 40% in microgravity [3,5]. In consequence, a change in the baroreceptor reflex as well as in organ blood supply and a dysregulation of the cerebrovascular system can be observed in weightlessness [3,6]. However, it remains unclear if the increase in perfusion compensates for a higher metabolic demand in weightlessness or if the peripheral resistance is too low for the human heart in microgravity conditions [7]. Given the observed adaptational changes in the heart, recent studies also speculate about the possibility of a reduction in cardiac performance due to the deconditioning and restructuring of the heart, thus inducing heart failure and, in consequence, potential arrhythmias [8]. In addition to these changes, long-term exposure to weightlessness was reported to cause a dysregulation of the immune system and an alteration of the microbiome, leading to the increased virulence of pathogens in weightlessness [7]. Furthermore, atrophic processes in bones and muscles are frequent findings after long-term exposure to weightlessness [9]. A recent investigation of our study group also observed a decrease in hemoglobin and an increase in the glomerular filtration rate (GFR) [10]. However, while the changes mentioned above have been described and analyzed extensively by numerous studies, further investigations of the molecular background of these processes remain scarce. 

Cardiac biomarkers have been studied extensively over recent years and represent novel and promising diagnostic tools in the assessment of cardiovascular disease entities. Especially in heart failure, biomarkers have been proven to have great potential regarding diagnosis and prognosis. In this context, a multimarker approach was reported as most effective due to the incorporation of different pathophysiological processes relevant to the cardiovascular system [11]. Among them, the heart-type fatty acid binding protein (H-FABP—myocardial ischemia), soluble suppression of tumorigenicity 2 (sST-2—myocardial strain/stress and inflammation) and its ligand interleukin-33 (IL-33—inflammation), growth differentiation factor-15 (GDF-15—inflammation, remodelling), soluble urokinase-type plasminogen activator receptor (suPAR—inflammation, remodelling) and Fetuin-A (vascular calcification) have shown promising results in prior studies and have also found clinical application in the treatment of heart failure and cardiovascular disease [12,13,14,15,16].

Accordingly, given the lack of information on the molecular background of physiologic changes in response to weightlessness, we sought to perform a head-to-head analysis of these six heart failure biomarkers in humans undergoing parabolic flight as a spaceflight analogue. Thereby, we aimed to better understand the molecular mechanisms and cardiac involvement in these adaptational processes and thus enlighten the topic of weightlessness-induced heart failure.

## 2. Results

In total, this study included 14 healthy volunteers (eight males) with a mean age of 28.9 years. The detailed baseline characteristics for all 14 volunteers are presented in Table 1. 

### 2.1. Biomarker Levels

#### 2.1.1. sST2

**Figure 1:**
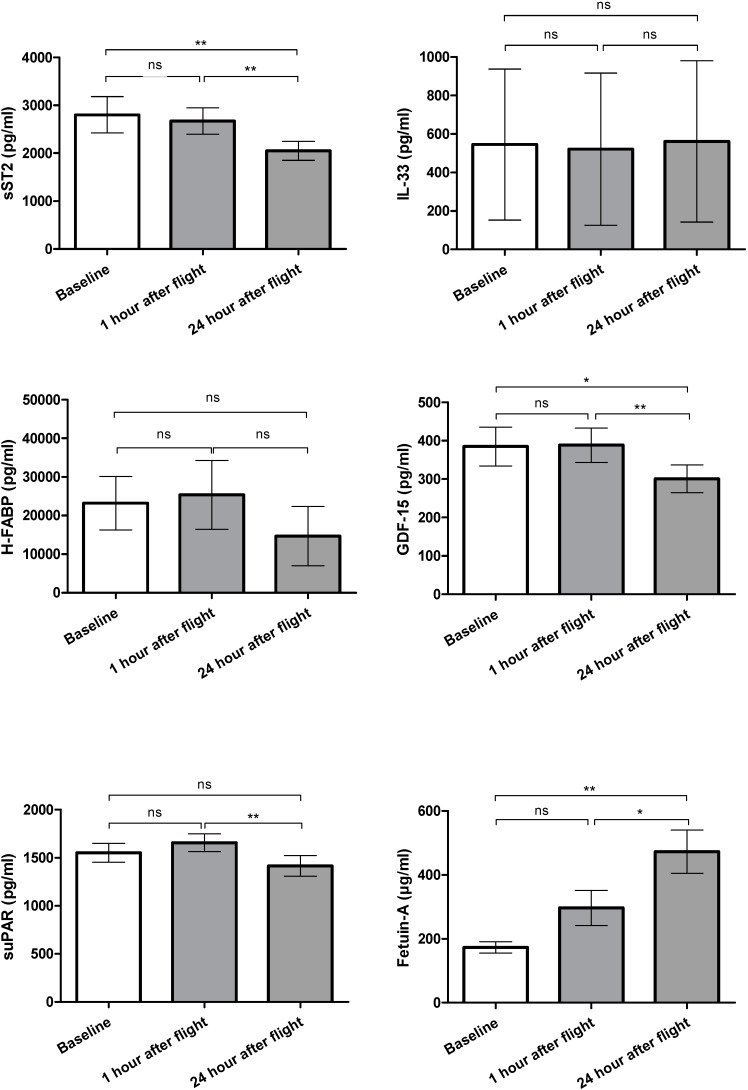
Comparison of biomarker levels at baseline/1 h after/24 h after parabolic flight (ns = not significant, * *p* < 0.05, ** *p* <0.01).

**Figure 2:**
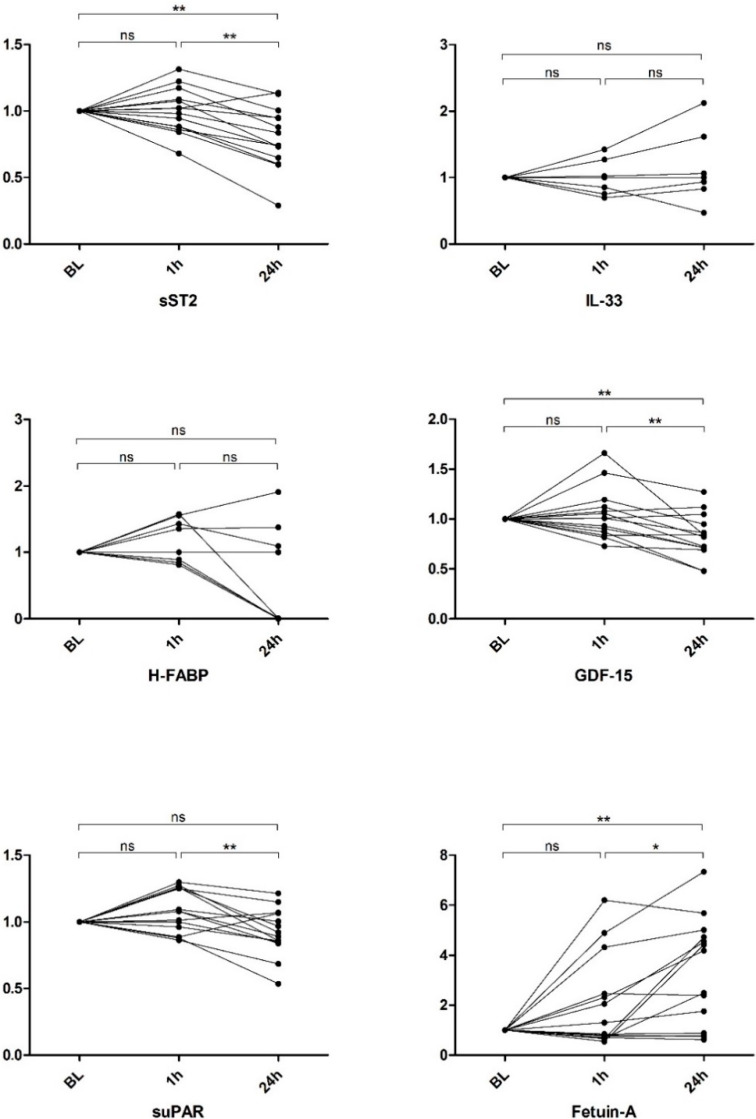
Fold change analysis of biomarkers at 1 h after/24 h after parabolic flight (ns = not significant, * *p* < 0.05, ** *p* <0.01).

The serum levels of sST2 remained unchanged 1 h after parabolic flight (2672 pg/mL; SEM 276 pg/mL) compared to the baseline levels (2800 pg/mL; SEM 380 pg/mL, *p* = 0.760). By 24 h after parabolic flight (2050 pg/mL; SEM 199 pg/mL), a significant decrease in sST2 was found compared to the values at baseline and at 1 h after parabolic flight (*p* = 0.009 and *p* = 0.004, respectively; see Figure 1). This finding was consistent in the fold change analysis (baseline vs. 1 h, *p* = 1.0; baseline vs. 24 h, *p* = 0.0085; 1 h vs. 24 h, *p* = 0.006. See Figure 2).

#### 2.1.2. IL-33 

The serum levels of IL-33 were 545 pg/mL (SEM 392.0 pg/mL) at baseline, 521 pg/mL (SEM 396.0 pg/mL) after 1 h and 561 pg/mL (SEM 419.0 pg/mL) after 24 h. There were no significant differences between the respective timepoints (baseline vs. 1 h, *p* = 1.0; baseline vs. 24 h, *p* = 0.6875; 1 h vs. 24 h, *p* = 0.3750. See Figure 1). This finding was also consistent in the fold change analysis (baseline vs. 1 h, *p* = 1.0; baseline vs. 24 h, *p* = 0.3750; 1 h vs. 24 h, *p* = 0.1563. See Figure 2).

#### 2.1.3. H-FABP

The H-FABP levels remained without any significant changes and were 23,000 pg/mL (SEM 7000 pg/mL) at baseline, 25,000 pg/mL (SEM 9000) at one hour and 15,000 ng/mL (SEM 8000 ng/mL) at 24 h after parabolic flight (baseline vs. 1 h, *p* = 0.625; baseline vs. 24 h, *p* = 0.3223; 1 h vs. 24 h, *p* = 0.0977. See Figure 1). This finding was also consistent in the fold change analysis (baseline vs. 1 h, *p* = 0.1094; baseline vs. 24 h, *p* = 0.2188; 1 h vs. 24 h, *p* = 0.0547. See Figure 2).

#### 2.1.4. GDF-15 

The GDF-15 levels were 385 pg/mL (SEM 51 pg/mL) at baseline and remained without significant changes 1 h after parabolic flight (389 pg/mL; SEM 45 pg/mL, *p* = 0.9515). At 24 h after parabolic flight (301 pg/mL; SEM 36 pg/mL), a significant decrease was evident (baseline vs. 24 h, *p* = 0.002; 1 h vs. 24 h, *p* = 0.025. See Figure 1). Similar findings were evident in the fold change analysis (baseline vs. 1 h, *p*= 0.8552; baseline vs. 24 h, *p* = 0.0085; 1 h vs. 24 h, *p* = 0.0031. See Figure 2).

#### 2.1.5. suPAR 

The suPAR levels did not evidence significant changes one hour after parabolic flight compared to the baseline values (1552 pg/mL; SEM 97 pg/mL and 1658 pg/mL; SEM 94 pg/mL, *p* = 0.1099). The levels at 24 h after parabolic flight were significantly decreased (1415 pg/mL; SEM 107 pg/mL, *p* = 0.009)) compared to the values at 1 h, while no significant changes were apparent compared to the baseline values (*p* = 0.1726; see Figure 1). This finding was also consistent in the fold change analysis (baseline vs. 1 h, *p* = 0.1272; baseline vs. 24 h, *p* = 0.1531; 1 h vs. 24 h, *p* = 0.004. See Figure 2).

#### 2.1.6. Fetuin-A 

The Fetuin-A levels were 173 µg/mL (SEM 18 µg/mL) at baseline without a significant change at one hour after parabolic flight (297 µg/mL; SEM 55 µg/mL, *p* = 0.3). By 24 h after parabolic flight, a significant increase in Fetuin-A was evident compared to the levels at baseline (473 µg/mL; SEM 68 µg/mL, *p* = 0.007) and the levels at one hour after parabolic flight (*p* = 0.04; see Figure 1). Likewise, the fold change analysis evidenced a significant increase in Fetuin-A levels at 24 h after parabolic flight compared to the levels at baseline and 1 h (baseline vs. 1 h, *p* = 0.1937; baseline vs. 24 h, *p* = 0.0052; 1 h vs. 24 h, *p* = 0.0419. See Figure 2).

### 2.2. Correlation Analysis

In a correlation analysis regarding the baseline characteristics, we found no relevant correlations between the biomarker expression and baseline characteristics. Likewise, the correlation of biomarker expression and laboratory parameters shown in the supplemental table showed no consistent correlations over all three time points. Of note, the GDF-15 levels (*p* = 0.03) and sST2 levels (*p* = 0.007) were associated with higher CRP levels at baseline. Additionally, myoglobin showed a significant correlation with the IL-33 levels (*p* = 0.025) and H-FABP levels (*p* = 0.01) at 24 h. A detailed correlation analysis is given in Table S1. There were no significant differences between male and female volunteers regarding the biomarker levels and laboratory parameters (Table S2). 

## 3. Discussion

Along with a growing focus on human space missions, space medicine as a prerequisite for safe and successful missions has gained significant awareness in recent years [3]. While different adaptational processes have been reported by numerous studies, cardiovascular complications still constitute the most important issues encountered during space travel [3,9]. The latest studies have also reported a potential deconditioning and restructuring of the heart, leading to heart failure [8]. However, the molecular mechanisms behind these findings remain largely unknown, thus giving rise to further investigations. Accordingly, to further analyze these adaptational processes and to address the topic of weightlessness-induced heart failure, we aimed for an analysis of heart failure biomarkers in response to parabolic flight as a spaceflight analogue.

Regarding the levels of sST2, we found a significant decrease 24 h after parabolic flight, while no changes were observed one hour after parabolic flight. While the membrane-bound ST2L receptor mediates cardioprotection by the binding of IL -33, sST2 acts as a decoy receptor for IL-33, thus preventing its (beneficial) effects [12]. Additionally, the correlation of the baseline levels of sST2 and CRP points out its suspected involvement in inflammatory processes [12]. sST2 is associated with increased cardiac strain and cardiac fibrosis and is elevated in clinical scenarios of heart failure and acute coronary syndrome [17]. Vice versa, the decrease in sST2 observed in our study could constitute an indicator of a reduction in cardiac stress and strain [12]. Our findings match at least in part former studies that speculate about an influence of microgravity on cardiac stress and cardiac strain. Among others, Iskovitz et al. reported a reduction in cardiac stress in microgravity, while the left ventricular strain distributions where not significantly different in different gravitational fields [8]. Additionally, an increase in left atrial and left ventricular volume was reported [18]. Together with an increase in cardiac output of up to 40% in zero gravity, as reported in former studies, a reduction in cardiac stress may seem contradictory [19]. However, cardioprotective effects, such as a reduction in heart rate as well as a decrease in peripheral vascular resistance, have been observed in weightlessness, similar to the therapeutic effects of heart failure treatment in the clinical setting [19]. Accordingly, these effects seem to surpass the cardiac stress induced by the increased pre-load volume in zero gravity. However, a recent study proposed a decrease in cardiac performance by means of the deconditioning and restructuring of the heart in response to the reduction in cardiac stress [8].

Interestingly, no significant changes were observed regarding the levels of IL-33 in our study. However, a trend towards higher levels was evident 24 h after parabolic flight. Considering that IL-33 represents the only known ligand for the ST2-receptor, an IL-33-independent effect induced by sST2 seems unlikely [20]. Accordingly, a delay in the increase in IL-33 in response to the decrease in its decoy receptor seems the most probable explanation for the lack of dynamic in IL-33. This is mainly attributed to the small time period in which the biomarker measurements were conducted. 

Besides its cardioprotective effects, IL-33 is involved in numerous immunologic processes. It is responsible for immunomodulation, influencing the secretion and interaction of a range of immune defense cells, particularly T helper 2 (TH2) cells, mast cells, group 2 innate lymphoid cells, (ILC2s), regulatory T (Treg) cells, TH1 cells, CD8+ T cells and natural killer (NK) cells [21,22]. However, the interaction between these cell types and the role of IL-33 is not fully understood yet. Nevertheless, given the immunomodulatory role of IL-33, a decrease in sST2 might also be involved in the alterations of the immune system in weightlessness described in previous studies [23]. An essential role of IL-33 in allergic diseases, such as bronchial asthma and atopic dermatitis, further highlights the immunomodulatory effects of IL-33 and thus also of weightlessness [22]. Accordingly, the ST2/IL-33 interaction offers a promising target for space medicine in the future.

In contrast, the H-FABP levels did not show any significant changes at 1 h and 24 h after parabolic flight. H-FABP constitutes a highly sensitive marker of myocardial ischemia and myocardial damage, providing a sensitivity even superior to troponin [24]. Accordingly, these findings again indicate no increase in cardiac stress in response to gravity changes. Furthermore, based on these results, it seems that the myocardial oxygen demand is not critically elevated in response to gravitational changes. Of note, considering the minimal secretion of H-FABP in non-ischemic cells together with our young and healthy study collective, a potential decrease in H-FABP levels in response to gravitational changes must be assumed as not detectable. This finding also suits a former study of our working group on the influence of parabolic flight on different blood parameters. Similar to H-FABP, we could not detect any significant changes in Troponin and BNP in response to parabolic flight [10]. This again emphasizes the assumption that no significant myocardial ischemia is induced by the gravitational changes encountered in parabolic flight. The levels of GDF-15 were significantly decreased at 1 h and showed a further decline at 24 h after parabolic flight. Accordingly, given its involvement in the response to cardiac stress and myocardial ischemia as well as coronary artery disease, the observed decrease in GDF-15 further supports the assumption of a reduction in cardiac stress and ischemia in response to microgravity [14]. However, as GDF-15 is also involved in inflammatory processes and the regulation of apoptosis and remodeling, it seems that the exposure to weightlessness could also have an additional influence on processes regarding the cell cycle and immune system, as was also proposed by recent studies [7,25]. Accordingly, the correlation of the GDF-15 levels at baseline with the CRP levels in our analyses matches former studies. In this regard, a recent study also reported an involvement of GDF-15 in the modulation of transcriptional regulation in the Smad pathway [25].

This theory is further confirmed by analyzing the levels of suPAR, which showed a significant decrease 24 h after parabolic flight, without any significant changes 1 h after parabolic flight. suPAR is a reliable indicator for the activity level of the immune and inflammatory system and correlates with organ damage in diverse organ systems, including the cardiovascular system [15,26]. Thus, the decrease in suPAR in response to zero gravity seems to indicate an anti-inflammatory effect accompanied by a decrease in the activity of the immune system. This finding matches the results of prior studies, describing an impairment of the immune system in zero gravity [7].

In contrast to the markers discussed above, Fetuin-A showed a significant increase 24 h after exposure to parabolic flight. Given the involvement of Fetuin-A in calcium homeostasis and vascular and tissue calcification, a decrease in free calcium levels resulting in a reduction in extraosseous calcification with a calcium shift towards the intraosseous departments could be suspected [16]. However, this finding represents a contradiction to the reports of osteoporotic processes in astronauts after long-term exposure to weightlessness in former studies [9]. Since our study proposes a possible reduction in extraosseous calcification processes at least in response to short-term gravitational changes, a change in calcium-homeostasis between short- and long-term exposures should be considered. Of note, metabolic diseases known to interfere with the secretion of Fetuin-A (e.g., diabetes, CKD) were ruled out in all study subjects. 

Another important point with regard to our results is the biological variability of the tested biomarkers, since different timepoints were defined for the analysis of these markers. Of note, investigations into the circadian dynamics of GDF-15, suPAR, H-FABP and the sST2/IL-33 pathway were conducted in former studies [27,28,29,30,31,32]. Except for suPAR, which did not show a diurnal secretion pattern, a circadian dynamic was reported, with an interval of about 12 h between the peak and nadir in the majority of cases. Of note, the differences observed with regards to a circadian dynamic are lower than the changes observed in response to parabolic flight. While research on a potential circadian dynamic of Fetuin-A is still a matter for further investigation, recent studies have shown a stable expression pattern without significant changes over 72 h [33]. Accordingly, given the timepoints of blood-sampling with measurements at 5 h and 24 h after the baseline measurements, as well as the predominantly clockwise dynamic with comparably low circadian changes in biomarker levels, a difference in diurnal secretion patterns seems unlikely as a relevant confounder of our findings. Thus, with respect to the secretion profile of our tested heart failure biomarkers, we found molecular correlates for several weightlessness-dependent changes described in former studies. Above all, similar to former investigations, we suspect a decrease in cardiac stress, which might be most probably based on the decrease in peripheral vascular resistance and heart rate observed in zero-gravity. These findings are supported by studies that show a significant increase in cardiac biomarkers with long-term physical activity [34]. Moreover, studies showing cardiac atrophy with an up-to-12% reduction in left ventricular mass in weightlessness underline the significant decrease in cardiac stress in zero gravity [3]. Additionally, further investigations in simulated microgravity and computer models observed a potential reduction in cardiac stress [8]. Accordingly, in synopsis with our findings and former studies, both indicating a decrease in cardiac stress, a potential reduction in cardiac performance by means of deconditioning and restructuring of the heart in response to weightlessness has to be taken into account. In this regard, the potential development of heart failure in long-term exposure to weightlessness represents an important target for future studies. Furthermore, our data emphasize the potential influence of weightlessness on the immune system through impairment and a decrease in inflammatory activation, reflected in the reduction in inflammatory biomarkers [7]. Additionally, our findings propose a potential difference in calcium homeostasis between short- and long-term space missions. While osteoporotic changes are observed in long-term missions, based on our findings, a short-time exposure to weightlessness may have an opposite effect [9,16]. 

However, given the novelty and the hypothesis-generating approach of the present study, the possible practical application of our findings will remain a topic for further investigation. Nevertheless, heart failure biomarkers could be of great benefit in the course of the timely identification of people at risk for complications in zero gravity, similar to risk stratification in clinical medicine [35]. In this regard, a multimarker measurement seems the most promising approach. By incorporating different pathophysiological processes present in the response to gravitational changes, heart failure biomarkers could be of value as monitoring and safety parameters, particularly in the course of preventive medical care for crews on long-term space missions.

## 4. Materials and Methods 

### 4.1. Participants

The study was conducted in accordance with the Declaration of Helsinki (1975, revised in 2008) and the protocol was approved by the German Ethics Committee of the Medical Faculty of the University Hospital Duesseldorf, Germany (Date of approval: August 14th, 2017; Project Identification number: 2017054297) and by the French Ethics Committee (Comité de Protection des Personnes (CPP Nord-Ouest III) of the Medical Faculty of the University of Caen (Date of approval: 6 September 2017; Project Identification number: 2017-A01185-48). In total, we enrolled 14 healthy participants in this study. Recruitment was conducted at the University Hospital of Duesseldorf, Germany. All the participants signed a written informed consent form. The inclusion criteria were defined as: age >18 years, airworthiness, cardiorespiratory health, spontaneous circulation and signed informed consent. The exclusion criteria were defined as: a history of primary cardiovascular and respiratory diseases or the regular intake of medication, except for oral contraceptives; missing or withdrawal of informed consent; insufficient requirements for airworthiness; and a positive pregnancy test. Further details have been published in a study outline paper of our study group [36].

### 4.2. Parabolic Flight

**Figure 3:**
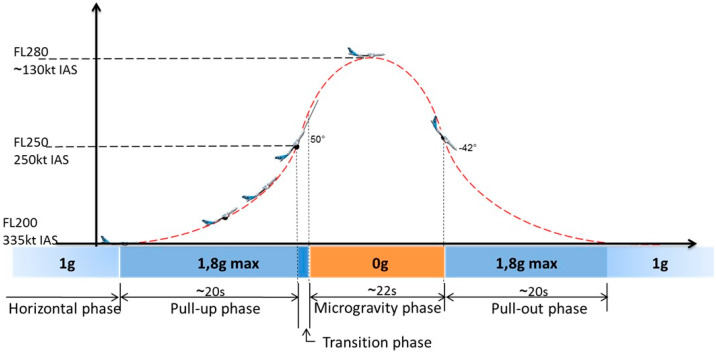
Illustration of a parabolic flight maneuver (copyright by NoveSpace).

A “Parabolic flight” represents a special aerial maneuver aimed at achieving a state of weightlessness (Figure 3). From a stabilized level-flight altitude (1 g), a steep ascent up to 47° is initiated by the pilots (1.8 g). After this climb, the so-called “injection” maneuver is performed. The power thrust is reduced, and the plane is directed into descent to follow the trajectory. During this phase, the vertical load factor shifts to zero gravity. With the plane tilting forward, the exit phase is initiated, leading to an up-to-45° descent and a second phase of hypergravity (1.8 g), before re-entering the steady flight (1 g) [37]. Each phase averages between 20 and 25 s, respectively [37].

### 4.3. Experimental Set-Up

The study was conducted as part of the 31st Parabolic Flight Campaign (PFC) of the German space agency (Deutsches Zentrum für Luft- und Raumfahrt-DLR), which took place from February 26th to March 11th, 2018, in Bordeaux, France. All flights were performed by the company NoveSpace (France, Mérignac). In total, four flight days were conducted in the course of the PFC, with one flight day consisting of 31 parabolas. Each study subject participated in one flight day and repetitive participation was avoided. Blood samples were drawn at three different time-points: 1 h prior to parabolic flight (baseline 08:00 ± 0.75 h), one hour after the parabolic flight (13:00 ± 1.0 h) and 24 h after the parabolic flight (08:00 ± 0.75 h) Serum samples were collected with individual punctures at each time point, using the following materials: BD vacutainer tubes (Becton Dickinson, Mountain View, CA, USA), 3 serum-separating tubes (SST) (Reference # 367957; tube size: 75 × 13 mm; draw volume: 3.5 mL) and 1 SST tube (Reference # 367955; tube size: 100 × 13 mm; draw volume: 5 mL). The serum sample aliquots were frozen and stored at −80 °C for further analysis after blood withdrawal.

### 4.4. Statistical Analysis

A statistical analysis was performed using GraphPad Prism software (GraphPad Software, USA) and SPSS (IBM SPSS Statistics, USA). Correlation analyses were conducted using Spearman’s Rank correlation. All data are given as mean ± standard error of the mean (SEM). The concentrations of biomarkers between time points were compared using the Wilcoxon matched-pairs test. To test the robustness of our findings, we also conducted a fold change analysis, again using the Wilcoxon matched-pairs test. *p*-values < 0.05 were considered statistically significant. A *p*-value of <0.05 was indicated as *, and a *p* of <0.01 as **. 

## 5. Conclusions

In conclusion, our findings indicate a decrease in cardiac stress, matching former studies. On this regard, a potential reduction in cardiac performance by means of deconditioning and restructuring of the heart in weightlessness must be taken into account. Furthermore, our data suggest an influence of weightlessness on the immune system through impairment and a decrease in inflammatory activation. Additionally, our findings propose a potential difference in calcium-homeostasis between short- and long-term exposure to weightlessness. However, given the novelty and the hypothesis-generating approach of the present study, further investigations on the adaptational processes are warranted.

## 6. Limitations

The biggest limitation of our study constitutes the small sample size, especially with regards to the biological and analytical variability of biomarkers. Nevertheless, the changes in biomarker levels were consistent in a fold change analysis, thus indicating robust results. When interpreting the findings of our study, one must keep in mind that the time frames of actual zero gravity were limited in the course of the parabolic flight maneuver. During an average flight day, the cumulative duration of weightlessness in total averaged 11 min and 20 s. Moreover, hypergravity must be considered as a potential confounder in parabolic flight. However, studies have shown a good comparability of short-time weightlessness by means of parabolic flight with space missions with regards to hemodynamic changes. Additionally, given the lack of previous analyses, no reference values are available in the literature. Thus, the hypothesis-generating character of this study must be emphasized. 

## Figures and Tables

**Table 1:** Baseline characteristics.

*n*	Sex	Age (y)	Height (m)	Weight (kg)	BMI (kg/m^2^)	BSA (m^2^)	BP Systolic (mmHg)	BP Mean (mmHg)	BP Diastolic (mmHg)	Heart Rate (bpm)
1	M	40	1.76	93	30	2.13	128	91	69	77
2	F	30	1.62	52	20	1.52	96	87	67	78
3	M	22	1.88	86	24	2.11	131	94	78	103
4	M	28	1.83	83	25	2.05	103	84	70	95
5	M	23	1.93	92	25	2.22	111	93	82	51
6	M	29	1.91	82	22	2.08	107	97	79	58
7	F	23	1.64	54	20	1.56	109	102	95	95
8	F	25	1.72	75	25	1.89	109	92	77	69
9	F	30	1.70	62	21	1.71	112	90	80	80
10	M	31	1.77	78	25	1.95	106	87	76	55
11	F	24	1.73	63	21	1.74	124	94	81	82
12	M	37	1.92	91	25	2.20	128	83	54	107
13	M	31	1.82	86	26	2.10	126	99	86	87
14	F	31	1.79	76	24	1.90	108	85	73	78
**Mean**	**m = 8**	**28.9**	**1.79**	**76.6**	**23.8**	**1.9**	**114.1**	**91.3**	**76.2**	**79.6**

## Supplementary Materials

Supplementary materials can be found at https://www.mdpi.com/1422-0067/21/10/3467/s1. Table S1: Correlation analysis of biomarker expression and baseline characteristics/laboratory parameters. Table S2: Analysis on gender related differences in biomarker levels.

## Abbreviations

CKD	chronic kidney disease
DLR	Deutsches Zentrum für Luft- und Raumfahrt
ELISA	enzyme-linked immunosorbent assay
g	gravitational force
GDF-15	growth differentiation factor-15
H-FABP	heart-type fatty acid binding protein
IL-33	interleukin-33
NASA	National Aeronautics and Space Administration
PFC	parabolic flight campaign
sST2	soluble suppression of tumorigenicity 2
suPAR	soluble urokinase-type plasminogen activator receptor
